# Numerical Simulation and Measurement of Deformation Wave Parameters by Sensors of Various Types

**DOI:** 10.3390/s23229215

**Published:** 2023-11-16

**Authors:** Nurzhigit Smailov, Sauletbek Koshkinbayev, Yerlan Tashtay, Ainur Kuttybayeva, Rimma Abdykadyrkyzy, Dmitry Arseniev, Dmitry Kiesewetter, Sergey Krivosheev, Sergey Magazinov, Victor Malyugin, Changsen Sun

**Affiliations:** 1 Department of Electronics, Telecommunications and Space Technologies, Satbayev University, Almaty 050013, Kazakhstan; n.smailov@satbayev.university (N.S.); sauke49@mail.ru (S.K.); y.tashtay@satbayev.university (Y.T.); ainur.kuttybayeva2023@gmail.com (A.K.); rizhanbayeva@mail.ru (R.A.); 2 Institute of Energy, Higher School of High Voltage Engineering, Peter the Great St. Petersburg Polytechnic University, St. Petersburg 195251, Russiaksi.mgd@gmail.com (S.K.); magazinov_sg@mail.ru (S.M.); vim@spbstu.ru (V.M.); 3 College of Optoelectronic Engineering and Instrumentation Science, Dalian University of Technology, Dalian 116024, China; suncs@dlut.edu.cn

**Keywords:** fiber Bragg grating, pulse elongation, diagnostics, high-speed deformation, sensors, strain gauge, spectrum

## Abstract

The results of applications of various methods for measuring the parameters of high-speed loading using a strain gauge, a fiber Bragg grating located on a metal measuring rod and an interferometer monitoring the movement of the free boundary of the end of the rod are presented. Numerical simulation confirmed the adequacy of the description of the shock-wave process according to experimental data and showed that, with the thickness of the adhesive layer fixing the fiber Bragg grating and the strain gauge on a dimensional rod up to 100 µm, the deformation parameters of the sensors correspond to the parameters of the stress–strain state of the rod. Experimentally, a good correspondence of the results of measuring the magnitude of the relative deformation at a pulse duration of 10–100 µs using sensors of various types is shown, and an estimate of the limit values of the measured values of the deformation wave parameters is given.

## 1. Introduction

The pulsed mechanical strength of materials significantly depends not only on such properties as the speed of sound, Young’s modulus, static tensile strength, temperature, etc., but also on loading modes. It is known that when testing a material in different loading schemes in shock-wave [1,2,3,4,5,6] and dynamic [7,8,9] modes and also in tests for dynamic crack resistance [10,11], an increase in destructive loads is observed with a decrease in the duration of exposure and a delay in destruction.

In particular, the efficiency of electrophysical and electric power equipment is determined by the design features and the set of strength characteristics of the materials used. At the same time, the mechanical destruction of a solid dielectric leads to a loss of electrical strength and a catastrophic change in the operating conditions of equipment elements. This situation fully applies to pulsed magnetic systems, the solid insulation in which is in an electrically and mechanically stressed state.

To create strong multi-component structures operating under pulsed and dynamic loads, it is necessary to take into account the characteristics of the properties of materials under these conditions. Identifying the features of deformation and destruction of materials requires monitoring the parameters of the acting pulse and the reaction of the material.

The measurement of the parameters of the fast-flowing processes of shock loading and deformation has its own specifics. So, in [12], the main measurement schemes used to register the parameters of shock-wave loading according to the open circuit are given. As the most accurate and inertia-free method of measuring small displacements, various schemes of interferometers are widely used in experiments on the pulsed loading of materials [13,14,15,16], the applicability of which has been demonstrated, including when testing materials loaded with controlled microsecond pressure pulses generated by the magnetopulse method [17].

An overview of some methods for high-rate deformation and shock studies is given in [18].

One of the widely used directions in the mechanics of a deformable body is tensometry. With the help of strain gauges, the parameters of shock-wave action and the reactions of materials are recorded when studying the deformation dependencies of various materials at deformation rates at the level of $10^{3}$ 1/s [19].

The use of interferometric measurement methods, as well as methods using strain gauges, despite the convenience and accuracy, is difficult in conditions of limited geometry of the deformable object and with a high level of electromagnetic interference. Other measurement methods, for example, those described in [20], also have their own scope. Fiber Bragg gratings (FBGs) are practically insensitive to strong electromagnetic fields. In particular, refs. [21,22] show the possibility of using fiber Bragg gratings to measure high-frequency vibrations of LaCoO${}_{3}$ in magnetic fields up to 600 T. Therefore, the study of the possibility of their application to measure the parameters of deformation waves is of particular interest for operating in conditions of strong pulsed electromagnetic interference.

The main purpose of this work is to substantiate the applicability of fiber Bragg gratings for measuring the parameters of the high-speed deformation of materials based on the comparison of data obtained using various experimental methods and the results of numerical simulations.

A large number of scientific papers have been devoted to the theory and practical use of FBG [23,24,25,26,27,28,29,30,31,32,33,34,35,36]. The main properties and principles of the operation of FBG are considered in reviews [23,24,25,26,34,37]; in particular, technology of FBG manufacturing is considered in [23,24,25,29,33,34,36,38], and solutions of applied problems are considered in [23,24,25,26,27,28,30,38,39,40]. The use of FBG for detecting and monitoring defects of concrete blocks is presented in [41], and mathematical solutions for some types of deformation are presented in [42].

In most cases, interrogators are used to determine the deformation parameters or temperature of FBG, which allows for the achievement of a high measurement accuracy but does not allow for the study of high-speed deformation. Systems for measuring the parameters of the high-speed deformation of objects using FBG are used quite rare and are not described in as much detail in the scientific literature as low-frequency systems. One of the directions is the shock-wave and detonation diagnostic systems described in [40,43,44,45,46,47,48]. The use of high-speed collecting for sounding in detonation and shock-wave experiments is considered in [37,46]. The determination of the shock wave parameters can be performed without destroying the FBG by measuring changes in the spectrum of reflected radiation in real time [47,48,49]. All these methods, as well as any other methods, have their advantages and disadvantages.

A simple alternative method for measuring the velocity stretching and compression of objects using FBG, but with a limited dynamic range of measurements, is described in [50]. In [50], the general principles of operation of such a sensor, the method of numerical modeling of the signal and an example of an application are also considered. It is shown in [50] that the power of radiation reflected from the FBG is uniquely related to the amount of elongation or compression of the FBG with relatively small impacts, but this dependence is nonlinear. Therefore, it is necessary to develop a signal processing technique to determine the magnitude of the relative deformation of an object.

Both strain gauges and sensors based on fiber Bragg gratings must have mechanical contact with the object under study. Usually, such sensors are attached to the surface of the object with glue (adhesive). It is known that the parameters of the adhesive layer affect the accuracy of measurements. This effect can be reduced by using more advanced sensor designs [51] for film load cells. When using a Bragg fiber grating made in a standard single-mode fiber, the only way to reduce the harmful effect of the adhesive layer on the measurement accuracy is the correct choice of the parameters of the adhesive joint. There are many works in which the results of the numerical modeling of the deformation of objects with Bragg fiber gratings attached to them are presented, such as [52]. However, additional research is needed to assess the effect of the adhesive layer on the measured parameters of the deformation wave, develop simple recommendations and achieve confidence in the reliability of experimentally measured values.

## 2. Modeling of Mechanical Waves in a Rod

The numerical simulation method allows us to investigate the effect of deformation waves on various types of sensors used, to evaluate the effect of the method of connecting the sensor to the object under study, in particular, the thickness and stiffness of the adhesive layer, on the wave parameters directly in the sensitive area of the sensor, which is difficult to perform experimentally. Therefore, the numerical simulation contains two parts, discussed below: modeling the deformation wave directly in the sensors and modeling the sensor signal under such an impact.

**Figure 1:**
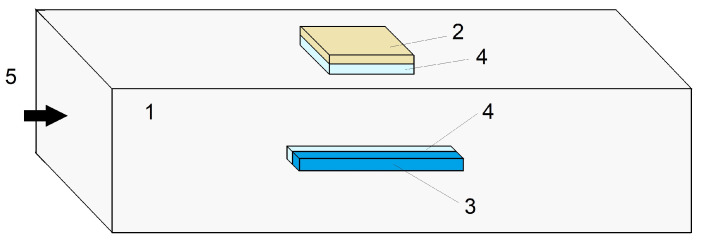
Schematic representation of a rod model with sensor elements used to simulate the propagation of a deformation wave (images are presented at different scales for clarity): 1—the rod, 2—phenolic substrate of the strain gauge, 3—the parallelepiped replacing an optical fiber during modeling, 4—layer of glue, 5—the direction of mechanical action launching the deformation wave.

The propagation of the compression wave along the rod (Figure 1), simulating the implementation of the Hopkinson method [18,53], was performed in the ANSYS AutoDyn environment. The optical cladding of the fiber, having a diameter of 125 µm, was replaced by a parallelepiped with a square cross-section (100 µm × 100 µm) made of the same material (i.e., fused quartz) as the cladding of the fiber to simplify modeling. In the middle of the aluminum measuring rod with the dimensions 1000 mm × 10 mm × 10 mm, the phenolic substrate of the strain gauge, which has dimensions of 5 mm × 6 mm × 0.1 mm, is located, along with a fused quartz element with dimensions of 10 mm × 0.1 mm × 0.1 mm that simulates an optical fiber, which is glued to the rod with a layer of glue, 0.1 mm thick. The following properties of materials were set:

- Aluminum measuring rod: Young’s modulus of 71 GPa, Poisson’s ratio of 0.33, and density of 2770 kg/m${}^{3}$;
- Phenolic substrate of the strain gauge: Young’s modulus of 32 GPa, Poisson’s ratio of 0.3, and density of 1800 kg/m${}^{3}$;
- Fused quartz parallelepiped: Young’s modulus pf 73 GPa, Poisson’s ratio of 0.2, and density of 2200 kg/m${}^{3}$;
- Glue: Young’s modulus varied in the range of 0.15–15 GPa, Poisson’s ratio of 0.3, and density of 1000 kg/m${}^{3}$.

The external action was set at the left end of the rod (Figure 1) in the form of the pressure pulse, described by the following expression:

(1) $$P=P_{m}{\left(\sin\left(\pi \frac{t}{T}\right)\exp\left(-\frac{t}{\tau }\right)\right)}^{2}$$

where $P_{m}=$ 14 MPa is the pressure amplitude, T= 50 µs is the half-wave duration and τ= 25 µs is the attenuation constant.

**Figure 2:**
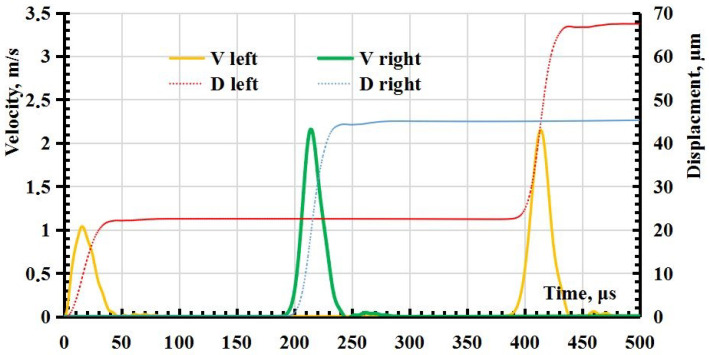
Dependence of displacement and speed of movement of the left and right ends of the rod (on the graph, *V* and *D* are the velocity and displacement on the right and left (see Figure 1)).

As a result of the calculation, the dependence of the displacement and the speed of movement of the ends of the rod are obtained (Figure 2), allowing the magnitude of the relative deformation at any given time to be determined. The speed of movement of the free end (right, Figure 1) of the rod ν, on which the mirror is applied in the experiment to register the displacements using the interferometer, is associated with relative deformations ϵ in the middle of the rod in the wave mode by the ratio [54] $\nu =2\epsilon \nu_{s}$, where $\nu_{s}$ is the speed of sound in the rod.

**Figure 3:**
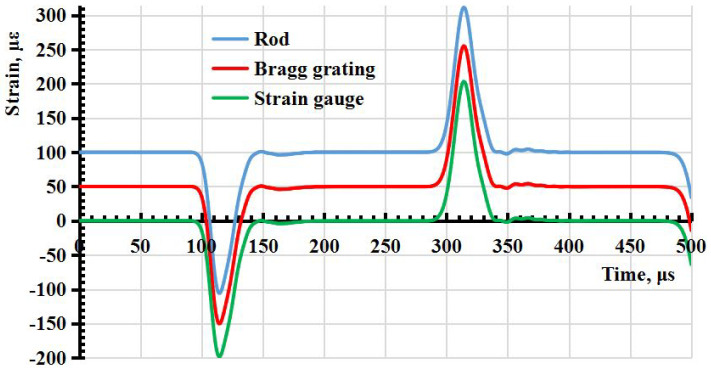
The dependence of relative deformations on time in the middle of the strain gauge, the fiber Bragg grating and the rod (the signals are shifted by $\epsilon =50\times 10^{-6}$ relative to each other).

It follows from the calculation results that the relative deformation is distributed unevenly over the elements simulating the surface of the strain gauge and fiber with Bragg grating, if assuming that the deformation in a parallelepiped is similar to the deformation in an optical fiber. The degree of unevenness depends on the elastic modulus and geometric dimensions of the strain gauge, the Bragg grating and the adhesive layer. Nevertheless, according to the calculation results, it can be seen that with the modulus of elasticity of the adhesive layer of 1.5 GPa in the middle of the strain gauge and the Bragg grating, the relative deformations coincide with the relative deformations on the surface of the rod both in amplitude and in the shape of the pulse (Figure 3).

**Figure 4:**
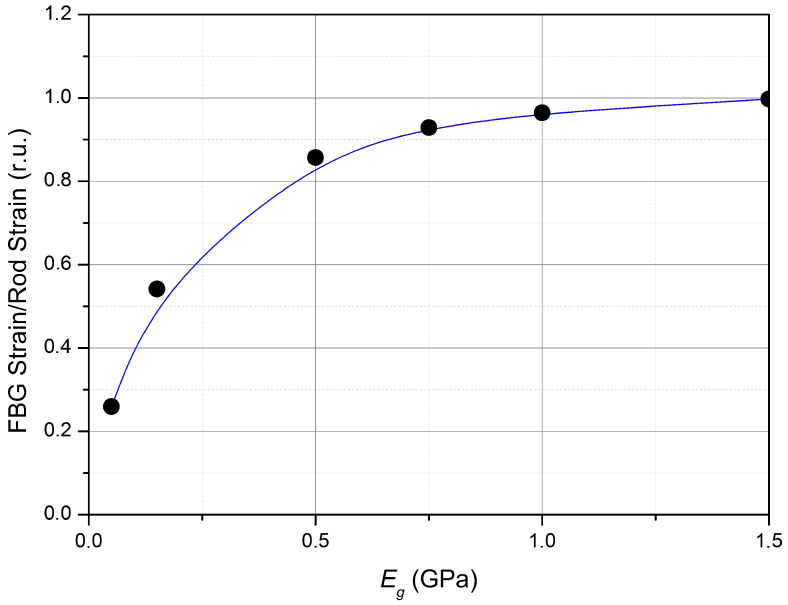
The dependence of the ratio of the strain in the fiber to the strain in the rod on the glue Young’s modulus (in relative units).

An example of the dependence of the ratio of the value of ϵ in an element simulating an optical fiber with a Bragg grating on the value of ϵ in the rod, that is, how many times the value of ϵ measured by the sensor differs from the true value of ϵ, is shown in Figure 4.

It follows from the results of numerical simulation that a decrease in the elastic modulus of the adhesive layer leads to an increase in the heterogeneity of the distribution of the relative deformations of the optical fiber and the strain gauge. For example, there is not only an increase in the inhomogeneity of the distribution but also a decrease in relative deformations in its middle by about 2 times in the case of an elastic modulus of 0.15 kPa in the Bragg grating of 10 mm length.

It can also be noted that an increase in the length of the glued part of the fiber to 100 mm, while maintaining the length of the Bragg grating equal to 10 mm, leads to a uniform distribution of relative deformations in the Bragg grating region, numerically coinciding with the relative deformations on the surface of the rod.

For the strain gauge with the above parameters, at the calculated values of the elastic modulus of the adhesive, the relative deformation of the sensor material did not differ much from the one in the rod.

Numerical simulation of the propagation of the deformation wave at different thicknesses of the adhesive layer between the sensors and the surface of the rod was also performed. In particular, when the thickness of the adhesive layer increases to 300 µm, the value of ϵ in the sensor is approximately 0.85 of the true value, and at 500 µm it is only 0.7 of the true value.

According to the simulation results, the correct choice of the elastic modulus of the adhesive and the thickness of the adhesive layer allow us to obtain the correct values of the measured value of the relative deformation of the rod and, more generally, of other objects.

## 3. Modeling of the Signal Caused by Reflection from the Fiber Bragg Grating

Numerical simulation of the signal caused by the reflection of radiation from FBG was performed in the Gaussian approximation of the radiation spectrum of a semiconductor laser and the reflection spectrum of FBG, similar to the work [50]. Let the pulsed mechanical action cause compression deformation with Gaussian dependence on time *t*:

(2) $$\epsilon \left(t\right)=A_{\epsilon }\exp\left(-{\left(\frac{t}{t_{h}}\right)}^{2}\right)$$

where $A_{\epsilon }$ is the proportionality coefficient and $t_{h}$ is the characteristic pulse time. In this case, an analytical solution can be obtained for the output signal.

**Figure 5:**
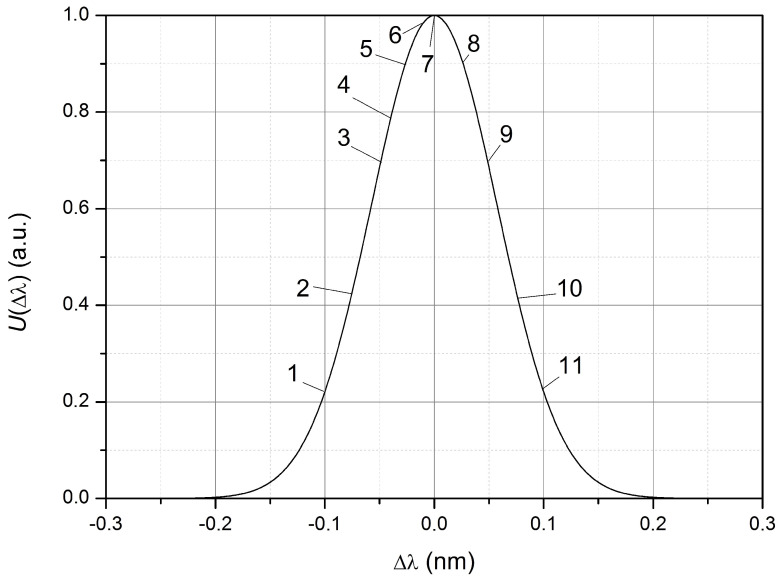
Dependence of the output signal on the difference in wavelengths in the absence of deformation of the FBG: 1–11—the position numbers of the working point for which the calculation U(t) was performed.

Then, the output voltage U(t) of the photodetector can be represented as follows [50]:

(3) $$U\left(t\right)=A_{a}\exp\left(-\left(\Delta \lambda +A_{\epsilon }k\exp\left(-{\left(\frac{t}{t_{h}}\right)}^{2}\right)\right)/{\sigma_{s}}^{2}\right)$$

where $\Delta \lambda =\lambda_{LD}-\lambda_{FBG0}$, $\lambda_{LD}$ is the central wavelength of the laser radiation at the operating temperature, $\lambda_{FBG0}$ is the resonant wavelength of FBG at the absence of deformation, *k* is the proportionality coefficient, $\sigma_{s}={\left({\left(\sigma_{LD}\right)}^{2}+{\left(\sigma_{FBG0}\right)}^{2}\right)}^{1/2}$ is the equivalent half-width of the spectrum in calculations and $A_{a}$ is the amplitude coefficient. The shape of the output pulse will depend on Δλ, i.e., the position of the working point. When FBG is compressed, the resonant wavelength of FBG decreases and Δλ increases. It follows from (3) that at ϵ=0 (i.e., in the absence of deformation) the dependence Δλ will also be Gaussian (Figure 5).

**Figure 6:**
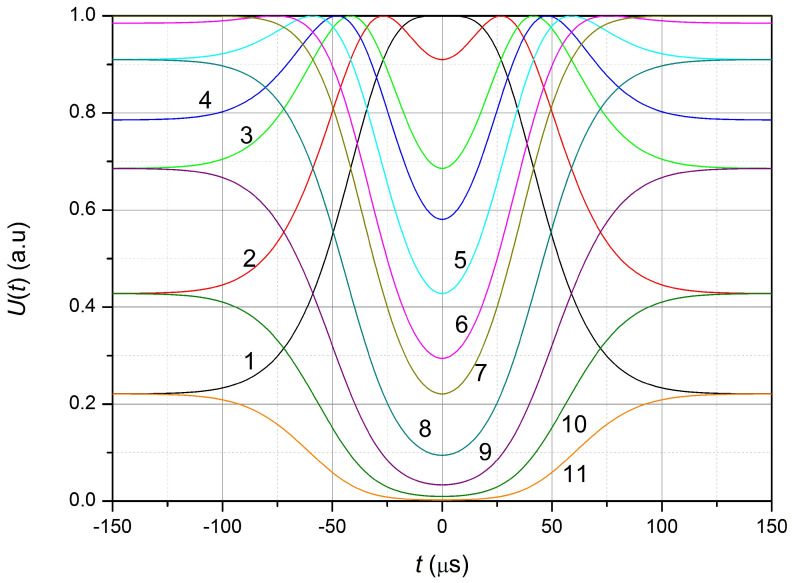
The calculated shape of the output pulse at the different positions of the working point (Δλ) in nm: 1—−0.1, 2—−0.075, 3—−0.05, 4—−0.04, 5—−0.025, 6—−0.01, 7—0, 8—0.025, 9—0.05, 10—0.075, 11—0.1.

Let us choose the value $t_{h}$ equal to 50 µs, which corresponds to the range of possible pulse durations used during the experimental study (10 …100 µs). The calculated shape of the output pulse for different operating points Δλ at $A_{\epsilon }$ equal to 100 µϵ is shown in Figure 6.

At operating point 1, compression of the FBG leads to an increase in the output signal, due to an increase in Δλ; i.e., the output signal is inverted with respect to the strain pulse of the FBG. At operating points 2–6 (Figure 6), when FBG is compressed, the signal reaches its maximum value at

(4) $$\Delta \lambda =A_{\epsilon }k\exp\left(-{\left(\frac{t}{t_{h}}\right)}^{2}\right))$$

and further decreases, due to an increase in the wavelength difference $\lambda_{LD}$ and $\lambda_{FBG}$. Therefore, the output voltage pulse has a minimum at time *t* = 0 (at the moment of the maximum relative compression of FBG). The greater the Δλ (at Δλ<0), the greater the voltage value in the signal minimum at t=0.

The output signal is not inverted with respect to the strain pulse of FBG at the working point Δλ>0 (points 8–11 in Figure 5 and Figure 6). However, in cases where the output signal approaches zero (points 10 and 11), the restoration of the shape and magnitude of the compression pulse (elongation) of the FBG, based on the received signal, is almost impossible near zero due to the influence of noise of the photodetector device.

For weak mechanical action, as shown in [50], the maximum sensitivity of the sensor is achieved at points

(5) $$\Delta \lambda =\pm \sigma_{s}/\sqrt{2}$$

The shape of the output signal will change, but all the patterns discussed above will remain for any other dependence (2) of the relative elongation on time.

## 4. Experimental Setup

**Figure 7:**
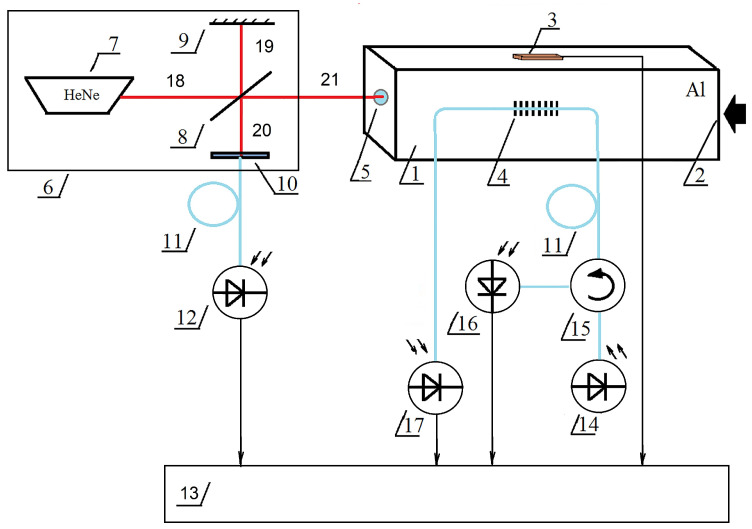
The scheme of the experimental setup: 1—square-section rod; 2—input end of the rod, which is mechanically affected; 3—strain gauge; 4—fiber Bragg grating; 5—mirror mounted on the output end of the rod; 6—Michelson interferometer (including the simplified scheme: 7—helium-neon laser; 8—translucent mirror; 9—fully reflecting mirror; 10—positioning device of the input end of the optical fiber); 11—optical fiber; 12—photodetector; 13—data conversion and processing device consisting of an amplifier, ADC and personal computer; 14—semiconductor laser mounted on the Peltier element; 15—optical circulator (can be replaced with a splitter); 16—photodetector converting radiation reflected by FBG; 17—photodetector converting radiation passed through FBG; 18–21—path of interfering beams.

The scheme of the experimental setup is shown in Figure 7.

The installation consists of the aluminum rod 1 with a rectangular cross-section of 10 mm × 10 mm and a length of 1 m; strain gauge sensor 3 (BF350-3AA) and fiber Bragg grating 4 glued to the rod at the distance of 0.5 m from the input end; mirror 5 glued to the output end of the rod; and Michelson interferometer 6, the scheme of which is given in a simplified form. Optical radiation for operation in the FBG sensor was created by semiconductor laser diode 14 placed on the Peltier element. The radiation reflected from the FBG, going through circulator 15, was transformed by photo detector 16. The fiber-optic circulator can be replaced with a splitter if there is a reserve of radiation power. The radiation that passed through the FBG was transformed by photodetector 17 in order to control the integrity of the optical fiber with the Bragg grating.

The measurements were performed using FBG with the following parameters: the resonant wavelength $\lambda_{FBG0}$ at room temperature was 1309.64 nm, the width of the reflection spectrum of the FBG ($\sigma_{FBG0}$) was 0.08 nm, the length of the FBG was 10 mm, the wavelength of the semiconductor laser ($\lambda_{LD}$) was 1310.04 nm, the width of the radiation spectrum ($\sigma_{LD}$) was 0.015 nm, the temperature coefficient of the wavelength variation was 0.071 nm/${}^{\circ }$C and the wavelength of the interferometer laser was 632.8 nm (He-Ne).

**Figure 8:**
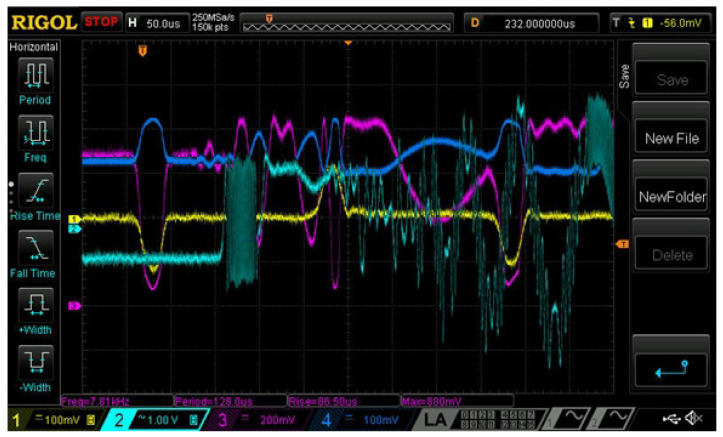
Screenshot from the oscilloscope screen. Waveform: 1 (yellow)—signal of the strain gauge, 2 (turquoise)—signal of the interferometer, 3 (purple)—radiation power reflected from the fiber Bragg grating, 4 (blue)—radiation power passed through the FBG. A legend is at the bottom of the screenshot. The scale on the axis 0X is 50 µs per division.

The shock action at end 2 launched a compression wave that propagated along the rod, also causing compression of the sensors used and, reaching the end of the rod with attached mirror 5, caused its displacement, which created an interferometer signal. The reflected wave, which is a stretching wave, propagated along the rod in the opposite direction. An example of a waveform obtained directly from the oscilloscope is shown in Figure 8.

## 5. Signal Processing

The interferometer signal arising from the interference of a beam reflected from a moving mirror with a reference beam is a sinusoidal oscillation with a varying period. It is known that the movement *S* of the mirror by a quarter of the wavelength ($\lambda_{I}$) of the interferometer light source radiation corresponds to a change in the signal from the minimum to the maximum value, i.e., half of the period. In this case, when the surface moves in one direction, the total displacement *S* for *N* half-periods is equal to $\left(N\lambda {}_{I}/4\right)$, and the total duration $t_{N}$ of *N* half-periods is calculated as follows:

(6) $$t_{N}=\sum_{i=1}^{N}\Delta t_{i}$$

where *i* is the summation index and $\Delta t_{i}$ is the time interval between extremes. The velocity ($v_{m}$) of the mirror movement in the time interval $\Delta t_{i}$ is calculated as $\lambda_{I}/\left(4\Delta t_{i}\right)$.

If we consider an interferometer signal with a zero mean value, then the time interval between two adjacent zero intersections corresponds to the time of the displacement of the mirror surface by the value $\lambda_{I}/4$. If the number of oscillations during the course of the process under study is large, then the dependence of the displacement of the mirror surface on time can be obtained by the simplest approximation from the intersection points of the zero value signal. Some refinement of the obtained dependence is given by taking into account the phase of the interferometer signal at the moment of the beginning of the mirror movement under the action of a compression or stretching wave.

The phase of interfering beams in the experimental setup is unstable due to the slow deformation of the interferometer structure with the rod under study. However, the characteristic time of these phase changes is several orders of magnitude less than when the mirror surface moves under the action of a compression wave. Therefore, a useful signal can be easily separated from a harmful effect.

One of the problems of signal processing is finding the reverse point at which the direction of the movement of the surface changes. However, in the case under consideration there is a unidirectional movement under the action of a compression wave. Therefore, this problem is not relevant for this task.

To obtain information about the parameters of the compression wave, the signal caused by the radiation reflected from the FBG also requires processing due to the nonlinear dependence of the signal amplitude on the magnitude of relative compression or elongation. When using the Gaussian approximation discussed above, when processing experimental data, it is important to correctly determine the following values: first, the conditionally zero level $U_{0}$ of the useful signal corresponding to the negligibly small reflection value FBG due to the reverse reflection from the docked fiber-optic elements, the dark leakage current of the photodetector and, possibly, the inaccurate zero setting of the photocurrent amplifier; secondly, the position of the working wavelength of the semiconductor laser relative to the spectral dependence of the reflection of the FBG; and thirdly, the value of the maximum possible signal FBG $U_{a}$, depending on the power of the semiconductor laser and the reflection coefficient of the FBG.

The required parameters can be obtained by changing the wavelength of the radiation of the semiconductor laser by heating and cooling it using a Peltier element. By choosing a temperature at which the laser wavelength is obviously outside the reflection range of FBG, the value $U_{0}$ can be measured. For standard fiber-optic elements with inclined (APC) polishing of the ends, the value of $U_{0}$ is negligible compared to the useful signal and it can be neglected.

The $U_{a}$ value corresponds to the maximum value of the signal caused by reflection from the FBG when the laser wavelength changes during heating and cooling at the absence of deformation of the FBG. In the Gaussian approximation, at $U_{0}=0$, the output signal can be calculated by the formula [50] corresponding to the Formula (3):

(7) $$U_{out}=U_{a}\exp\left(-\frac{{\left(\lambda_{FBG0}+k\epsilon -\lambda_{LD}\right)}^{2}}{\sigma_{s}^{2}}\right)$$

In the absence of stretching (or compression), i.e., at the initial state (at ϵ = 0), there is

(8) $$\frac{U_{out,i}}{U_{a}}=\exp\left(-\frac{{\left(\lambda_{FBG0}-\lambda_{LD}\right)}^{2}}{\sigma_{s}^{2}}\right)$$

It is possible to determine the difference in wavelengths $\Delta \lambda_{GL}=\lambda_{LD}-\lambda_{FBG0}$ or directly the wavelength $\lambda_{LD}$ by measuring the magnitude $U_{out,i}$ before the arrival of the deformation wave. Then, the relative elongation during the stretching (or compression) of FBG as a function of time $U_{out}\left(t\right)$ can be calculated by the following formula:

(9) $$\epsilon \left(t\right)=(-\sigma_{s}{\left(\ln\left(\frac{U_{out}}{U_{a}}\right)\right)}^{1/2}+\Delta \lambda_{GL})/k$$

The characteristic duration of the wavelength changing of FBG and laser due to temperature changes is significantly longer than the duration of the process under study. Therefore, during the execution of a single measurement of the waveform of the processes of propagation of compression and stretching waves, the difference in wavelengths can be assumed to be a constant value.

It is only necessary to perform an exact zero setting and correctly set the proportionality coefficient between the output voltage of the measuring device and the relative deformation to measure the deformation parameters using a strain gauge.

## 6. Data Obtained

As an example, consider the waveform shown in Figure 8. From the data obtained, it follows that $\sigma_{s}$ = 0.081 nm, $U_{a}$ = 880 mV and $U_{out,i}$ = 670 mV. Taking into account the parameters given above, the following can be obtained: $\Delta \lambda_{GL}$ = 0.0223 nm.

**Figure 9:**
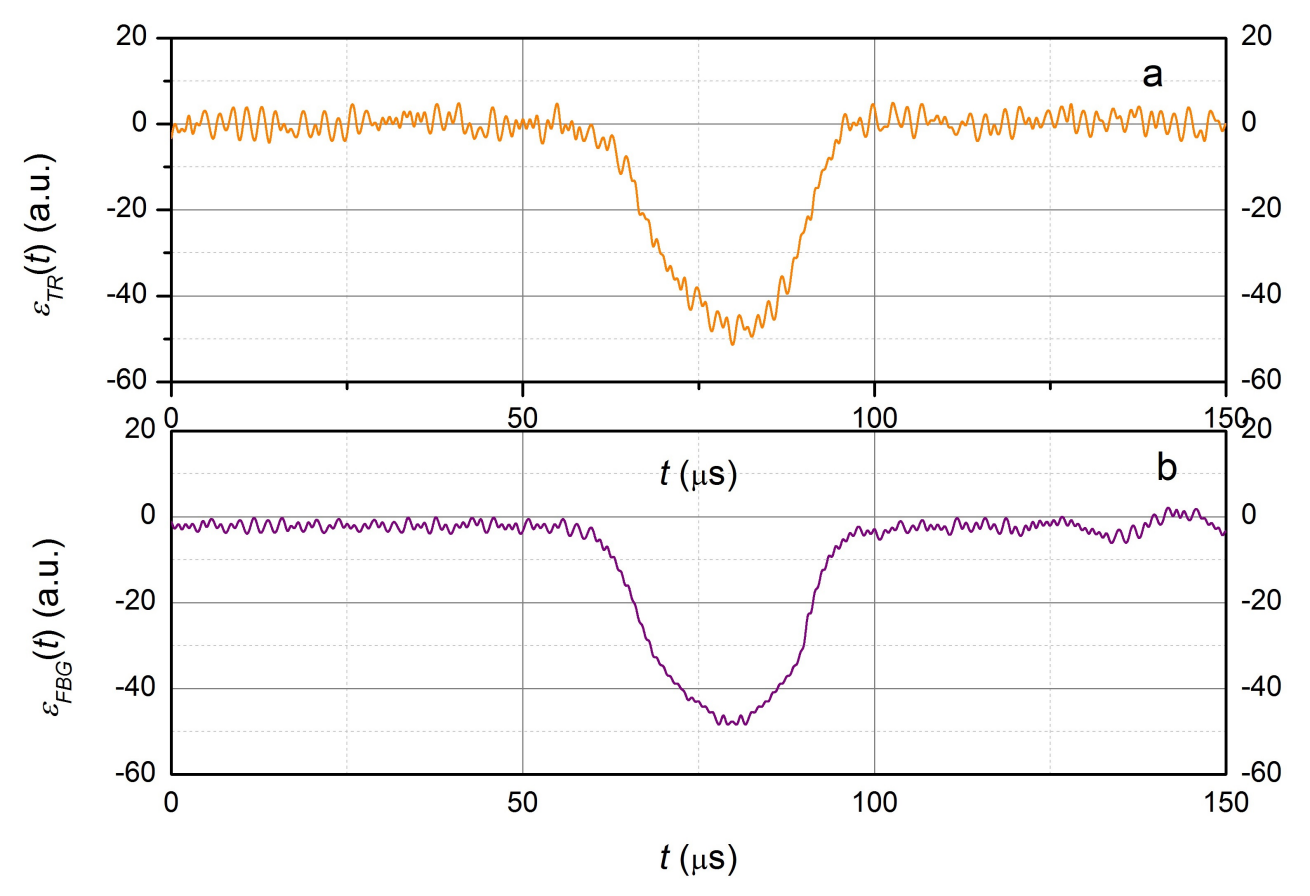
The experimentally obtained output signal from the tensor resistor (**a**) and the fiber Bragg grating after processing (**b**).

The value of the *k* coefficient is approximately equal to 1.2 × 10${}^{3}$ (or 1.2 × 10${}^{-3}$ nm per με) at the wavelength of 1550 nm [23,55]. The value of *k* at the operating wavelength can be obtained taking into account the direct proportionality of the coefficient *k* to the wavelength [55], i.e., at $\sigma_{LD}$, approximately equal to 1310 nm k≈ 1.0 × 10${}^{-3}$ nm per με occurs. The calculated dependence $\epsilon_{FBG}\left(t\right)$ obtained by Formula (9), as well as the value of the relative tension (compression) $\epsilon_{TR}\left(t\right)$ obtained using the strain gauge, is shown in Figure 9.

It follows from the presented data that the dependencies obtained using the FBG and the strain gauge are in good agreement.

It follows from Figure 8 also that the stretching wave reaching the output end of the rod causes the mirror to move at an average speed of approximately 0.6 m/s; the total displacement is approximately 20 µm. It should be noted that with the measurement method used there is no reverse point on the waveform of the interferometer signal, which indicates a unidirectional movement of the output end of the rod.

On the waveform shown in Figure 8, the signals caused by the reflected compression wave are clearly distinguishable—stretching waves (pulse of positive polarity) and repeated compression waves (negative polarity). These waves are also perceived by the FBG, but only the first pulse of the compression wave is clearly distinguishable. The signals of the FBG and interferometer, after the first compression wave reaches the output end of the rod, presumably, show the appearance of transverse vibrations that complicate signal processing. This is explained by the sensitivity of the FBG sensor to bends and the interferometer to mirror tilts. It can be assumed that the interferometer signal (approximately 100 µs after reaching the first compression wave of the output end) is due to both longitudinal and transverse vibrations of the rod, since the signal has two characteristic periods: approximately 50 µs and 3–5 μs. However, the period of these oscillations is unstable and strongly depends on the conditions for launching the stretching wave.

It should also be noted that when using an interferometer it is necessary to take into account the physical model of the propagation of the deformation wave in the rod to calculate the magnitude of the relative deformation of the material.

**Figure 10:**
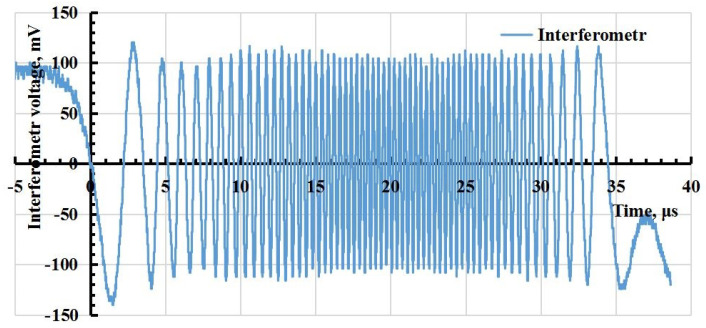
Waveform of the interferometer signal.

**Figure 11:**
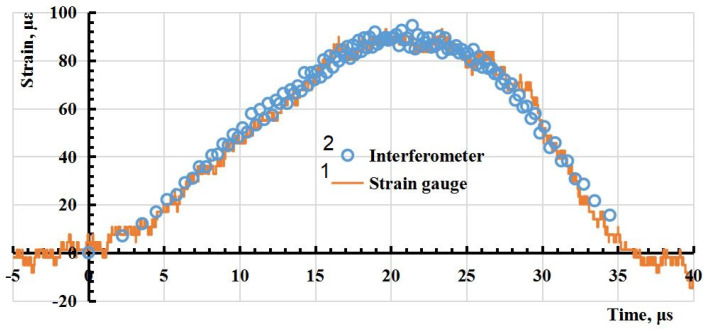
Comparison of the strain values obtained using the strain gauge (1) and the interferometer (2).

Waveforms of signals with a higher sampling rate of the oscilloscope ADC than those shown in Figure 10 were obtained for a more accurate calculation of the deformation value. The strain (relative deformation) of the rod ϵ, where the strain gauge is located, is proportional to the speed *v* of the free boundary of the rod, measured by the interferometer according to the equation $\epsilon =v/(2\nu_{s})$, where $\nu_{s}\approx$ 5000 m/s—the speed of sound in the aluminium rod. The resulting waveform of the interferometer signal is shown in Figure 10 and a comparison of the relative deformation values obtained using the interferometer and the strain gauge is shown in Figure 11. This suggests that there is a good correspondence of the measurement results performed by two different methods.

For compression wave pulses at the selected operating wavelength of the laser, the polarity of the signal pulse in FBG coincides with the polarity of the signal pulse of the strain gauge. For a stretching wave pulse, the signal of the FBG sensor at the pulse front increases, similar to the signal from the strain gauge, and after passing the point corresponding to the maximum $U_{FBG}(\epsilon$, when the resonant wavelength of the FBG and the wavelength of the laser radiation coincide) it decreases to almost zero. This corresponds to the theoretical concepts given in [55] and is also due to the position of the working wavelength of the laser near the maximum of the spectral dependence of the reflection of the FBG. Such a signal can also be processed and the dependence of the relative stretching on time can be obtained; however, presumably, transverse vibrations of the rod, which are difficult to take into account, reduce the accuracy of calculations.

**Figure 12:**
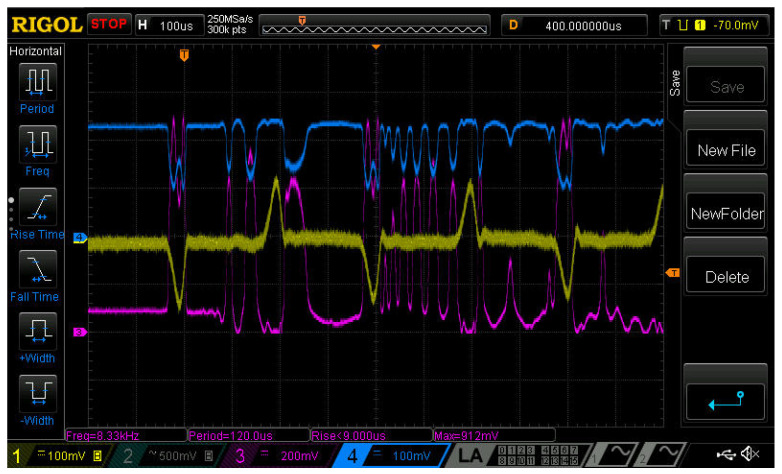
Waveform of signals (without the signal from the interferometer): 1 (yellow)—from the strain gauge, 2—not used, 3 (purple)—the signal caused by reflection from the FBG, 4 (blue)—the signal caused by radiation passed through FBG.

The dependence of $U_{FBG}\left(\epsilon \right)$ will be inverted with respect to $U_{TR}\left(\epsilon \right)$ when the working point (laser wavelength at ϵ = 0) is located on the declining part of the dependence U(Δλ) (for example, any of the points 8–11 in Figure 5). An example of such a waveform is shown in Figure 12. So, for a compression wave, the signal caused by reflection from the FBG has a positive polarity and a local minimum at the minimum value of the pulse signal of the strain gauge, indicating a transition through the extremum of the dependence U(Δλ) (Figure 5) near at the moment of maximum compression of the rod. The level of the $U_{FBG}\left(\epsilon \right)$ signal close to zero for the stretching wave is a consequence of the location of the working point U(Δλ) near zero (below point 11 in Figure 5).

## 7. Discussion

The results of the numerical simulation can be interpreted as follows. When exposed to the left end, a compression wave is formed in the rod. The compression wave reaches the right free end and is reflected by the stretching wave. That is, at the first perturbation, only a compression wave affects the left end, and a compression and stretching wave affect the right end, so the displacement and velocity of the right end is 2 times greater than the left. When a re-reflected wave from the right one comes to the left end, then the stretching wave will also act on the left end and then the compression wave and its velocity and displacement will also be twice as large as the primary displacement caused only by compression under external pressure (this can be seen in Figure 1 at a time of about 400 µs).

It can be assumed that the replacement of the cylindrical shape of an optical fiber (a rod with a circular cross-section) with a rectangular one (i.e., a rod with a rectangular cross-section made of quartz glass) does not significantly affect the simulation results, at least in cases where the mechanical stress in the glass rod is approximately equal to the stress in the measuring rod. So, for such a case, some changes in the parameters of the glue, the thickness of its layer and the size of the glass rod do not lead to a change in the design speed and the magnitude of deformation in the glass rod. If the shape of the cross-section of the light guide used in the simulation affects the calculation result, then, presumably, this can only be the case when the value of the maximum mechanical stress in the optical rod will significantly differ from the mechanical stress in the dimensional rod itself.

The model used also assumes that the mechanical parameters of the optical fiber cladding do not differ from these parameters of the fiber core with Bragg grating made in it, as well as the parameters of the quartz glass set during modeling.

It can also be assumed that the measurement technique is not significantly affected by either the measurement of the resonant wavelength of the FBG itself or the measuring rod and the adhesive layer when the temperature changes, although they all have different coefficients of thermal expansion, since the temperature change can be considered a slow process and it is assumed that the temperature effect is compensated by the movement of the working point, i.e., the wavelengths of a semiconductor laser.

The mechanical pulse of a rarefaction or compression wave in a real experiment, as well as in modeling, is not a Gaussian function. However, the use of the Gaussian approximation discussed above allows us to obtain analytical expressions for the output signal, which facilitates the analysis of the data obtained and allows us to describe the main patterns. The use of a Gaussian time function other than (4) affects only the shape of the pulse but does not change either the maximum value of the signal at the same maximum value of the stretching (compression) of the FBG or the principle of choosing the wavelength of a semiconductor laser (working point) for measurements.

The interferometer has the greatest sensitivity to longitudinal deformation. However, technically, it is difficult to place the reflecting mirror of the interferometer directly on the deforming surface, maintaining only sensitivity to longitudinal displacements and providing a weak influence of transverse displacements. It should also be noted that when moving the interferometer mirror at a distance significantly exceeding λ/2, the bandwidth of the photodetector device should be significantly greater than when using other types of sensors. The longitudinal displacement measured by the interferometer must be converted into the amount of mechanical deformation in the rod, using some specific physical model of the propagation of the compression or stretching wave—in the case under consideration, in a rectangular metal rod.

FBG-based strain sensors glued to the surface of the object under study allow you to measure the deformation parameters using the technique described above. The deformation dynamic measurement range of such a technique is limited, and the sensitivity is not adjustable, but the frequency range of thw measurements is limited only by the bandwidth of the photocurrent amplifier and can reach hundreds of megahertz. The dynamic range of measurements can be increased using a semiconductor laser, a super luminescent diode with a larger spectrum width or several FBGs with different resonant wavelengths.

## 8. Conclusions

The use of numerical modeling allowed us to prove that the adhesive layer gluing the sensors to the rod, with a layer thickness of 100 µm or less and a Young’s modulus of 1.5 GPa or more, as well as the sensors themselves, does not practically affect the accuracy of measuring the magnitude of deformation and the shape of the signal. That is, such a method of attaching sensors can be used when measuring the parameters of the deformation wave in the rod and determining the parameters of materials using the Hopkinson method, as well as, presumably, when studying the deformation waves of more complex objects. The correspondence of the measurement results of the deformation wave parameters found through three different methods (using the strain gauge, fiber Bragg grating and the interferometer) with a relative deformation value of up to 50 μϵ has been experimentally confirmed.

## Data Availability

Data are contained within the article.
